# Reoperation Versus Self‐Reported Recurrence After Ventral Hernia Repair: A Nationwide Survey‐ and Register‐Based Study

**DOI:** 10.1002/wjs.70512

**Published:** 2026-07-28

**Authors:** Evy Á Lakjuni Guttesen, Hugin Reistrup, Anders Gram‐Hanssen, Jacob Rosenberg, Jason Joe Baker

**Affiliations:** ^1^ Department of Surgery Center for Perioperative Optimization Copenhagen University Hospital—Herlev and Gentofte Herlev Denmark

**Keywords:** recurrence, reoperation, self‐reported recurrence, ventral hernia

## Abstract

**Background:**

Reoperation is often used as an outcome measure after ventral hernia repair but may underestimate the true recurrence rate, potentially leading to biased interpretation of studies using reoperation for recurrence as an outcome. This study aimed to determine the proportion of patients with recurrence after ventral hernia repair who undergo reoperation for recurrence in a nationwide population.

**Methods:**

This nationwide survey‐ and register‐based study, part of the AFTERHERNIA Project, included patients ≥ 18 years undergoing primary ventral or incisional hernia repair between January 2014 and March 2024. Patients were identified in the Danish National Patient Register and invited to participate in an electronic survey. Survey responses were linked to the Danish Ventral Hernia Database. Recurrence was defined as either reoperation for recurrence or self‐reported recurrence. Prevalences were estimated and adjusted for using regression analyses with marginal standardization.

**Results:**

A total of 20,703 patients were included (16,904 primary ventral and 3799 incisional hernias; 80% response rate), with a median follow‐up of 5.5 years. Among primary ventral hernias, overall recurrence was 15.5% (95% CI, 15.0–16.1), of which 3.3% (95% CI, 3.1–3.6) had a reoperation and 12.2% (95% CI, 11.7–12.7) had self‐reported recurrence. Among incisional hernias, overall recurrence was 27.2% (95% CI, 25.8–28.6), of which 7.0% (95% CI, 6.3–7.7) had a reoperation and 20.2% (95% CI, 18.9–21.5) had self‐reported recurrence.

**Conclusion:**

The prevalence of reoperation for recurrence underestimated the overall prevalence of recurrence by approximately fivefold after primary ventral hernia repair and fourfold after incisional hernia repair.

## Graphical abstract

Reoperation is often used as an outcome measure after ventral hernia repair but may underestimate the true recurrence rate, potentially leading to biased interpretation of studies using reoperation for recurrence as an outcome. This study aimed to determine the proportion of patients with recurrence after ventral hernia repair who undergo reoperation for recurrence in a nationwide population. The prevalence of reoperation for recurrence underestimated the overall prevalence of recurrence by approximately fivefold after primary ventral hernia repair and fourfold after incisional hernia repair.

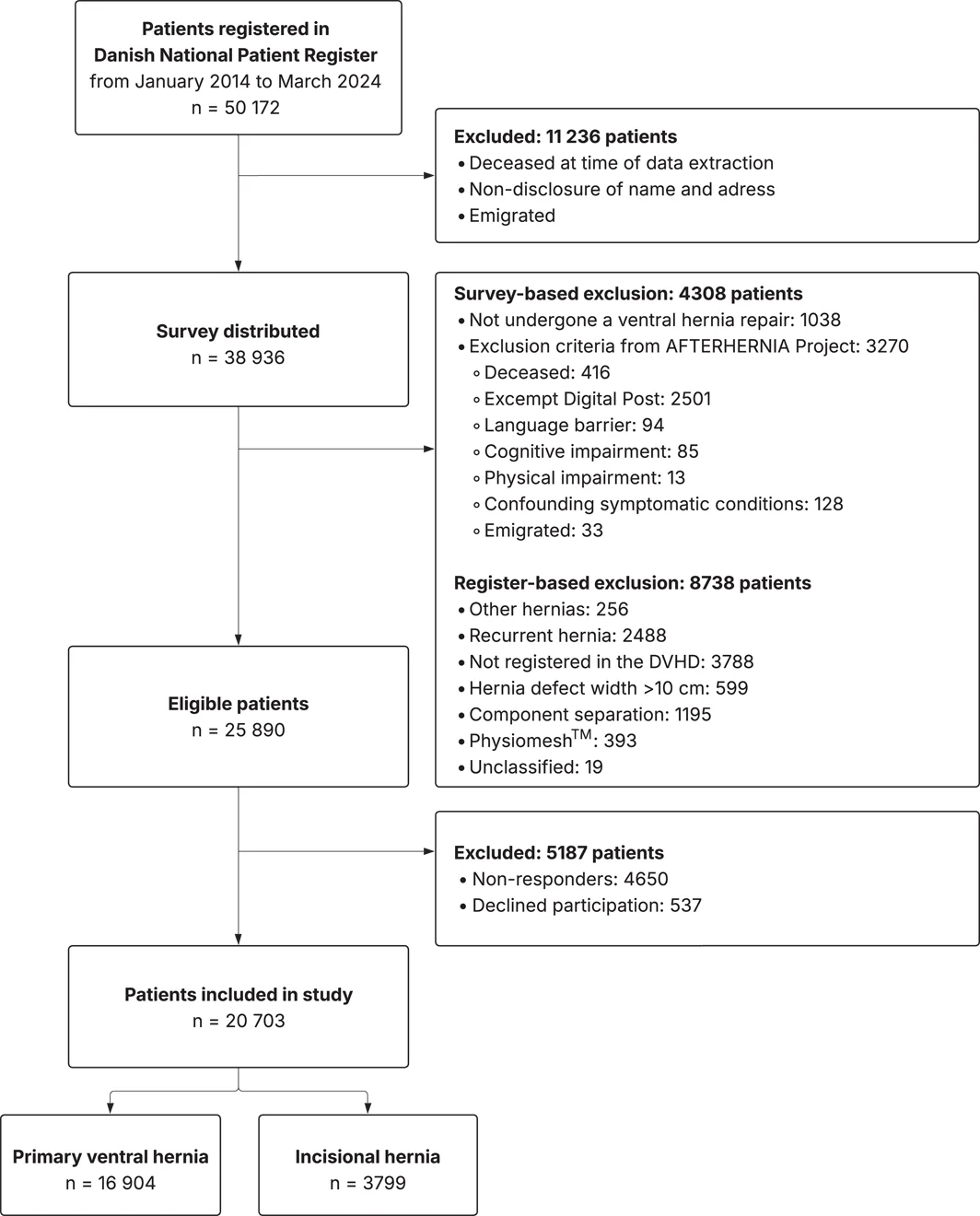

## 1 Introduction

Ventral hernia repair is among the most performed surgical procedures [1]. Evaluation of the technical success of the procedure is often assessed by recurrence. Recurrence may be estimated using several methods, including reoperation for recurrence, clinical examination, radiologic imaging, self‐reported recurrence, or combinations of these approaches [2, 3, 4, 5].

In registry‐based studies, reoperation for recurrence is frequently used as a proxy for recurrence because it is objective and readily available [6, 7, 8]. However, a previous prospective cohort study including 902 patients comparing reoperation with clinical recurrence demonstrated that reoperation substantially underestimated the overall risk of recurrence [4]. Moreover, radiological evaluation may even identify additional recurrences not detected by clinical examination [9], and these radiologically detected defects are not always symptomatic [10]. This finding suggests that a considerable proportion of patients experience recurrence without undergoing reoperation. In recent years, there has been an increasing emphasis on incorporating patient‐reported outcomes (PROs) into the evaluation of surgical success [11, 12]. Because reoperation captures only a subset of recurrences, PROs provide important insight into recurrences that do not lead to further surgery after ventral hernia repair.

We hypothesized that reoperation for recurrence underestimates the true burden of recurrence after ventral hernia repair. Therefore, this study aimed to compare reoperation for recurrence with overall recurrence, defined as either reoperation for recurrence or self‐reported recurrence, after ventral hernia repair on a nationwide scale.

## 2 Methods

### 2.1 Study Design

This nationwide survey‐ and register‐based study was based on prospectively collected data from the Danish Ventral Hernia Database [13] and survey data collected in the AFTERHERNIA Project [14]. The study was reported according to the Reporting of Studies Conducted Using Observational Routinely Collected Health Data (RECORD) statement [15] and the Consensus‐Based Checklist for Reporting of Survey Studies (CROSS) [16].

### 2.2 Setting

All patients undergoing ventral hernia repair in Denmark from January 1, 2014, to March 31, 2024, were identified via the Danish National Patient Register [17]. Eligible patients were subsequently invited to participate in an electronic survey via Digital Post [18]. Digital Post is a secure national platform for communication between citizens and public authorities in Denmark, and it is used by 95% of individuals aged 15 years or older [19]. The survey responses were further linked to the Danish Ventral Hernia Database, which includes data on patients' characteristics and operative details [20]. Further details on the general survey methodology are available in the AFTERHERNIA Project protocol [14].

### 2.3 Registries

All residents in Denmark are assigned a personal identification number, which is used in all interactions with both the private and public healthcare systems and enables linkage between national registers [21]. In the AFTERHERNIA Project, patients were identified through the Danish National Patient Register [17] using procedure codes from the Nordic Medico‐Statistical Committee Classification of Surgical Procedures (NOMESCO), indicating ventral hernia repair [22]. Using the personal identification number, person‐level data were further linked to the Danish Civil Registration System, which provides continuously updated information on emigration and vital status [21]. Additional linkage was performed with the Danish Ventral Hernia Database [13], a national clinical quality register that has prospectively collected data since 2007 on patients undergoing ventral hernia repair in both public and private healthcare sectors. The database provides detailed information on patients' demographics, hernia characteristics, and operative details entered directly by the operating surgeons [20]. Registration of all hernia repairs is mandatory in Denmark. According to the most recent annual report, the Danish Ventral Hernia Database captures about 82% of all ventral hernia repairs performed in Denmark [23], and has been validated against medical records with variable‐specific accuracies ranging from 89% to 99% [24].

### 2.4 Survey

The AFTERHERNIA Project uses an extensive questionnaire that includes questions on reasons for surgery, whether they had experienced a recurrence, lifestyle factors, and the Abdominal Hernia‐Q (AHQ) [25]. The questionnaire has been translated and validated for use in a Danish‐speaking population [26]. The present study used a single item from this questionnaire addressing whether patients suspected a recurrence after their hernia repair. The question was “Do you suspect that your hernia has returned since your most recent hernia operation?”. Response options were “yes” or “no”. The survey distribution began in August 2024, and responses were collected between August 2024 and September 2025. The survey was administered as a closed survey through the secure Research Electronic Data Capture (REDCap) platform [27], with each participant receiving a unique personal link. Non‐responders received up to three electronic reminders at 1‐week intervals. Patients who did not respond to these reminders were subsequently contacted by telephone and encouraged to participate. Approximately 70% of non‐responders had an active telephone number in the electronic patient record.

### 2.5 Participants

Eligible participants were adults (≥ 18 years) who underwent suture or mesh repair for a primary ventral hernia (umbilical or epigastric hernia) or an incisional hernia in Denmark between January 1, 2014, and March 31, 2024. Both elective and emergency repairs were included. Separate exclusion criteria were applied for the survey‐ and register‐based parts of the study. Survey‐based exclusions included patients who were deceased or had emigrated, were exempt from Digital Post, lacked sufficient Danish language skills, or were unable to complete the questionnaire due to cognitive deficits (e.g., dementia) or physical impairments (e.g., blindness or paralysis). Patients who felt unable to respond because of comorbidities or concurrent surgery, including disseminated abdominal malignancy or additional abdominal procedures that made it impossible to determine whether symptoms were related to the hernia repair, were also excluded. In addition, patients with undisclosed addresses in the Danish Civil Registration System and patients who denied having undergone ventral hernia repair were excluded. Register‐based exclusions included patients with recurrent hernia. Recurrent hernias were identified using a lookback period based on data from the Danish National Patient Register covering the years 1998–2014, which were included in the data supplied by the Danish Health Quality Institute, or if the first hernia repair was registered as a recurrence repair in the Danish Ventral Hernia Database. Additional exclusion criteria were procedures not registered in the Danish Ventral Hernia Database, a hernia defect width greater than 10 cm, use of Physiomesh [28, 29], and component separation procedures [30]. Follow‐up was defined as the time from index repair to reoperation for recurrence or survey response.

### 2.6 Outcome

The outcome of this study was the proportion of patients who underwent reoperation for recurrence compared with overall recurrence, defined as either reoperation for recurrence or self‐reported recurrence. Reoperation for recurrence was defined as a reoperation of the same type of hernia as the index repair. Self‐reported recurrence was assessed as a PRO using a single survey item with response options “yes” or “no”.

### 2.7 Data Sources & Variables Definitions

Data were extracted from the Danish Ventral Hernia Database and included sex, age, type of hernia, hernia defect width, Charlson Comorbidity Index, use of mesh, and mesh placement. Additional data on body mass index (BMI) and smoking status were obtained from the AFTERHERNIA Project questionnaire. Hernia defect width was categorized into ≤ 1 cm, > 1–4 cm, and > 4–10 cm in accordance with current guidelines [31]. BMI was categorized according to the Centers for Disease Control and Prevention definitions [32]. The Charlson Comorbidity Index [33] was grouped as 0, 1, 2, 3, and ≥ 4, with scores ≥ 4 combined. The type of repair was classified as suture or mesh repair. Mesh repair was further categorized by anatomical placement as onlay, retromuscular, preperitoneal, intraperitoneal onlay mesh without defect closure (IPOM), intraperitoneal onlay mesh with defect closure (IPOM plus), or other, including inlay and plug. As this was a nationwide register‐based study including all eligible patients, no a priori sample size calculation was performed.

### 2.8 Statistics

Categorical data were presented as counts and percentages, and continuous data were visually assessed with histograms and Q‐Q plots and presented as appropriate. Patients were divided into two distinct study groups based on hernia type: primary ventral hernias (umbilical and epigastric) and incisional hernias, which were analyzed separately [34]. The prevalence of recurrence for each group was analyzed with two different analyses. In the first analysis, patients with reoperation for recurrence or self‐reported recurrence were compared with those without recurrence. In the second analysis, patients with recurrence (overall recurrence), including both reoperation for recurrence and self‐reported recurrence, were compared with those without recurrence. In the first analysis, the adjusted prevalence of self‐reported recurrence and reoperation for recurrence was estimated simultaneously using multinomial logistic regression. To obtain population‐average adjusted prevalence estimates, marginal standardization (G‐computation) was applied. In the multinomial model, no recurrence served as the reference outcome group. In the second analysis, the adjusted prevalence of overall recurrence was estimated using multivariable logistic regression because the outcome was binary. Marginal standardization was likewise applied to derive population‐average adjusted prevalence estimates. Both models were adjusted for age [35], sex [35], BMI [35], smoking status [35], Charlson Comorbidity Index [33], hernia defect width [36], use of mesh [35], and follow‐up time (days) [34]. To account for the potential non‐linear association between follow‐up time and recurrence, follow‐up time was modeled using natural cubic splines (three degrees of freedom) [37]. Results were presented as prevalence percentages with 95% confidence intervals (CI). Analyses were based on complete cases without imputation.

All data cleaning and analyses were conducted in R version 4.5.0 [38]. Multinomial logistic regression and marginal standardization were performed using the *nnet* [39] and *marginaleffects* [40] packages. Within each group, four prespecified subgroup analyses were performed: three stratified by hernia defect width (small ≤ 1 cm, medium > 1–4 cm, and large 4–10 cm) and one restricted to patients aged < 65 years [35] with a Charlson Comorbidity Index score of 0. In addition, a post‐hoc analysis investigated crude rates for reoperation for recurrence among responders and non‐responders, including both primary ventral and incisional hernias.

### 2.9 Ethical Considerations

Informed consent was collected from all patients at the time of survey participation. This study obtained approvals from the Danish Health Quality Institute and the Danish Data Protection Agency (p‐2023‐14805). Ethical approval was not required under Danish legislation for the survey‐ and register‐based components of the study [41, 42]; ethical approval was, however, obtained from the Capital Region of Denmark's Committees on Health Research Ethics (R‐24033183) to access telephone numbers for reminder calls to non‐responders. The data were supplied by the Danish Health Quality Institute and the Danish Health Data Authority (FSEID‐00006834).

## 3 Results

### 3.1 Selection of Participants

**FIGURE 1:**
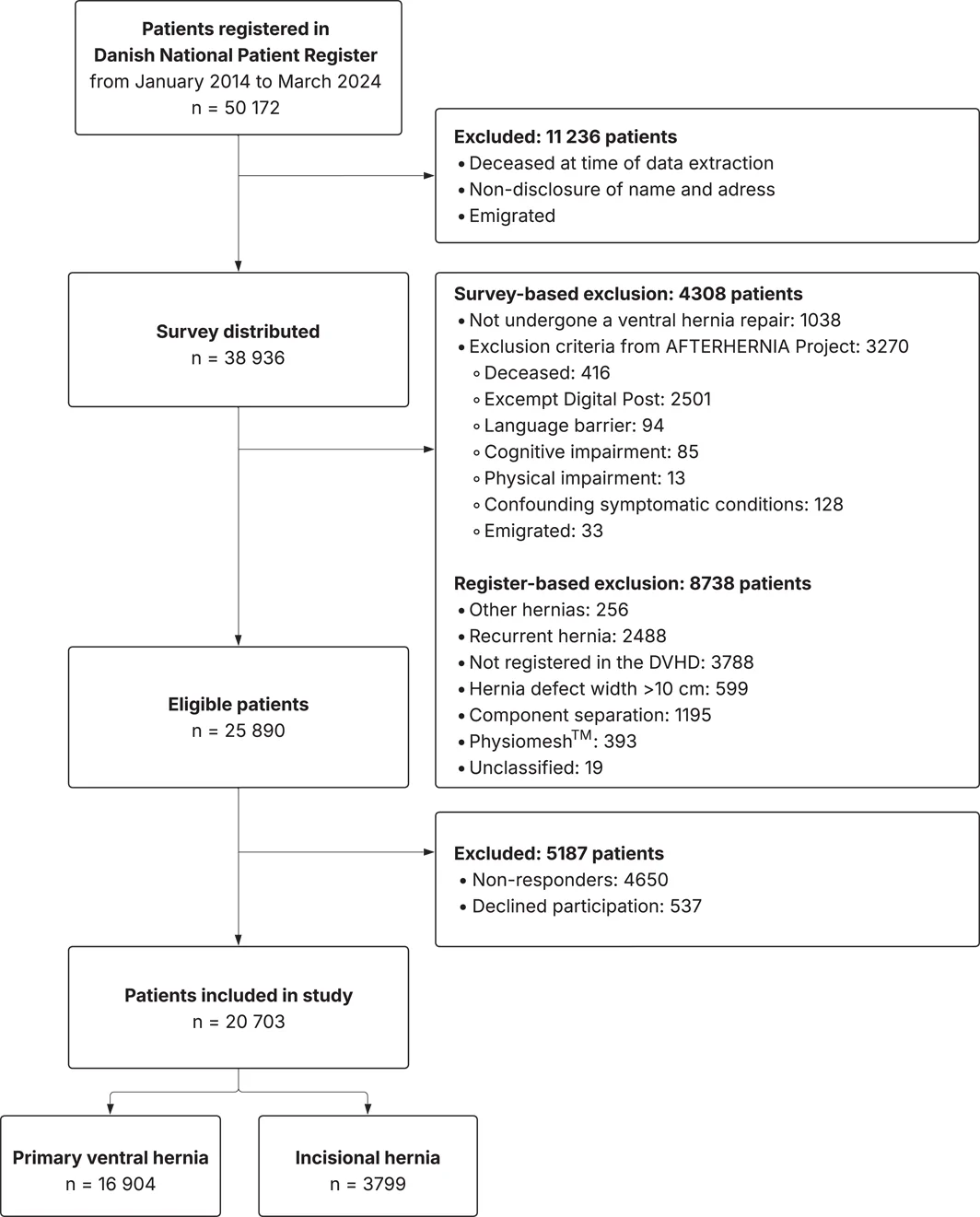
Flowchart of inclusion and exclusion. DVHD, Danish Ventral Hernia Database.

A total of 38,936 patients were invited to participate in the AFTERHERNIA Project (Figure 1), of whom 25,890 were eligible for inclusion after study‐specific eligibility criteria were applied. Overall, 20,703 patients responded to the survey, corresponding to a response rate of 80%. Among responders, 16,904 patients had primary ventral hernia, and 3799 patients had incisional hernia. The median follow‐up was 5.5 years (IQR, 2.9–7.9).

### 3.2 Patient and Operative Characteristics

Across both primary ventral and incisional hernias, patients with reoperation for recurrence and self‐reported recurrence were slightly younger compared with patients with no recurrence (Table 1). Patients with self‐reported recurrence had the longest follow‐up, whereas those undergoing reoperation for recurrence had the shortest follow‐up. In both groups, suture repair was more frequently used among patients who later underwent reoperation for recurrence, whereas mesh repair, particularly onlay, preperitoneal, and retromuscular mesh placement, was more common among patients with no recurrence. The distribution of BMI, smoking status, Charlson Comorbidity Index, and hernia defect width was otherwise similar across recurrence groups. Compared with patients with primary ventral hernias, patients with incisional hernias had greater comorbidity and larger hernia defect sizes. Patient characteristics and operative details were similar between responders and non‐responders (Supporting Information S1: Table 1), although non‐responders were slightly younger. Reoperation for recurrence occurred at similar proportions among responders and non‐responders when stratified by hernia type.

**TABLE 1:** Patient characteristics and operative details of primary ventral and incisional hernia stratified by recurrence status.

	Primary ventral hernia			Incisional hernia		
	No recurrence (*n* = 14,270)	Self‐reported (*n* = 2076)	Reoperation (*n* = 558)	No recurrence (*n* = 2766)	Self‐reported (*n* = 767)	Reoperation (*n* = 266)
Male sex	9953 (70)	1078 (52)	315 (56)	1494 (54)	479 (63)	148 (56)
Age in years, median [IQR]	53 [44–62]	48 [37–57]	48 [38–58]	59 [49–68]	57 [46–66]	56 [47–67]
BMI in kg/m^2^						
< 18.5	92 (1)	19 (1)	3 (< 1)	16 (< 1)	3 (< 1)	6 (2)
18.5–24.9	3595 (25)	604 (29)	155 (28)	672 (24)	182 (24)	54 (20)
25–29.9	5963 (41)	718 (35)	216 (39)	1125 (41)	267 (35)	106 (40)
30–34.9	3118 (22)	460 (22)	120 (22)	625 (23)	183 (24)	67 (25)
35–39.9	944 (7)	169 (8)	41 (7)	189 (7)	80 (10)	18 (7)
≥ 40	417 (3)	77 (4)	20 (4)	100 (4)	41 (5)	11 (4)
Missing	141 (1)	29 (1)	3 (< 1)	39 (1)	11 (1)	4 (2)
Smoking status						
Never	8514 (60)	1175 (57)	331 (59)	1538 (56)	391 (51)	135 (51)
Active	1983 (14)	382 (18)	89 (16)	437 (16)	151 (20)	37 (14)
Former	3742 (26)	512 (25)	136 (25)	780 (28)	222 (29)	94 (35)
Missing	31 (< 1)	7 (< 1)	2 (< 1)	11 (< 1)	3 (< 1)	0 (0)
Charlson comorbidity index						
0	10,775 (76)	1591 (76)	425 (76)	1412 (51)	380 (50)	125 (47)
1	1896 (13)	284 (14)	82 (15)	370 (14)	119 (15)	40 (15)
2	1038 (7)	121 (6)	34 (6)	609 (22)	154 (20)	60 (22)
3	307 (2)	41 (2)	9 (2)	177 (6)	63 (8)	15 (6)
≥ 4	254 (2)	39 (2)	8 (1)	198 (7)	51 (7)	26 (10)
Hernia defect width in cm						
≤ 1	7099 (50)	1190 (57)	337 (60)	495 (18)	151 (20)	54 (20)
> 1–4	6796 (47)	830 (40)	208 (38)	1486 (54)	379 (49)	128 (48)
> 4–10	375 (3)	56 (3)	13 (2)	785 (28)	237 (31)	84 (32)
Suture or mesh placement						
Suture	4428 (31)	919 (44)	299 (54)	230 (8)	78 (10)	57 (22)
Onlay	4850 (34)	533 (26)	102 (18)	641 (23)	197 (26)	63 (24)
Retromuscular	609 (4)	70 (3)	15 (3)	569 (21)	147 (19)	38 (14)
Preperitoneal	2272 (16)	230 (11)	46 (8)	347 (13)	68 (9)	11 (4)
IPOM	791 (6)	140 (7)	50 (9)	320 (12)	114 (15)	41 (15)
IPOM plus	1253 (9)	174 (8)	40 (7)	646 (23)	156 (20)	51 (19)
Other^a^	67 (< 1)	10 (1)	6 (1)	13 (0)	7 (1)	5 (2)
Follow‐up in years, median [IQR]	5 [3–8]	6 [4–8]	1 [1–4]	6 [4–8]	6 [4–8]	2 [1–4]

*Note:* Data are presented as *n* (%) unless otherwise indicated.

Abbreviations: BMI, body mass index; IPOM, intraperitoneal onlay mesh; IPOM plus, intraperitoneal onlay mesh with defect closure; IQR, interquartile range.

^a^ Other includes plug, inlay, and other mesh placements.

### 3.3 Adjusted Prevalence of Recurrence

**FIGURE 2:**
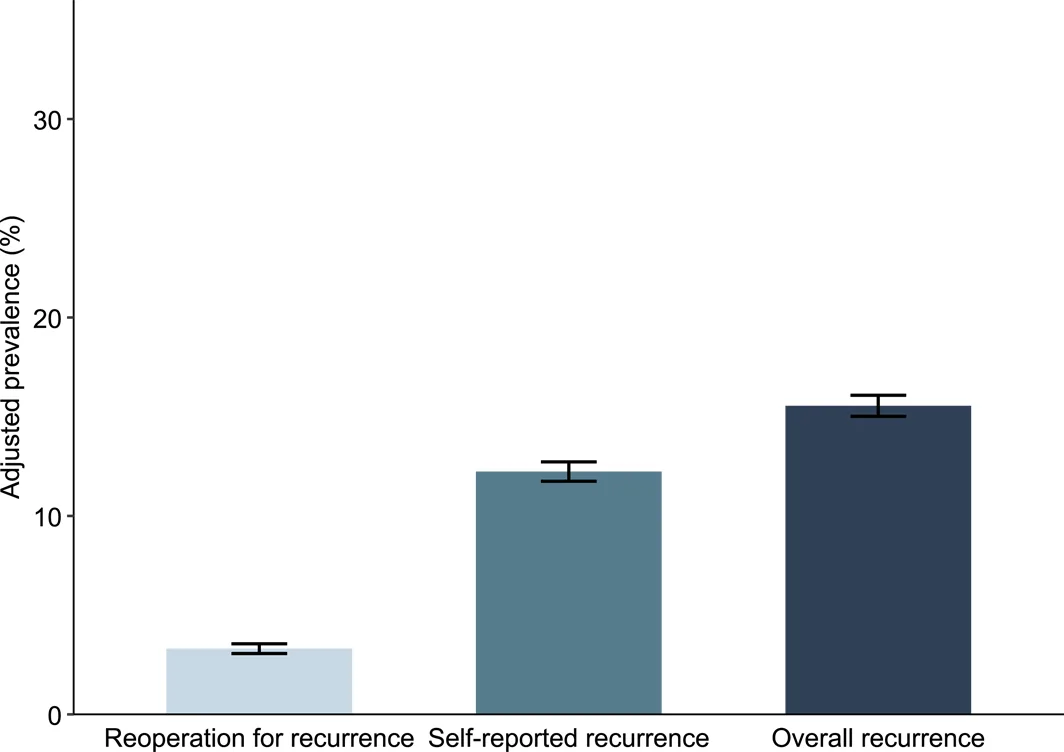
Primary ventral hernia repair. Adjusted prevalence of reoperation for recurrence, self‐reported recurrence, and overall recurrence (reoperation for recurrence or self‐reported recurrence). The adjusted prevalences were adjusted for sex, age, smoking, body mass index, Charlson Comorbidity Index, use of mesh, hernia defect width, and follow‐up. Error bars indicate 95% confidence intervals.

For primary ventral hernias, the adjusted prevalence of overall recurrence (reoperation for recurrence or self‐reported recurrence) was 15.5% (95% CI, 15.0–16.1), of which 3.3% (95% CI, 3.1–3.6) had a reoperation and 12.2% (95% CI, 11.7–12.7) had self‐reported recurrence (Figure 2). Thus, approximately one in five patients with primary ventral hernia recurrence underwent reoperation.

**FIGURE 3:**
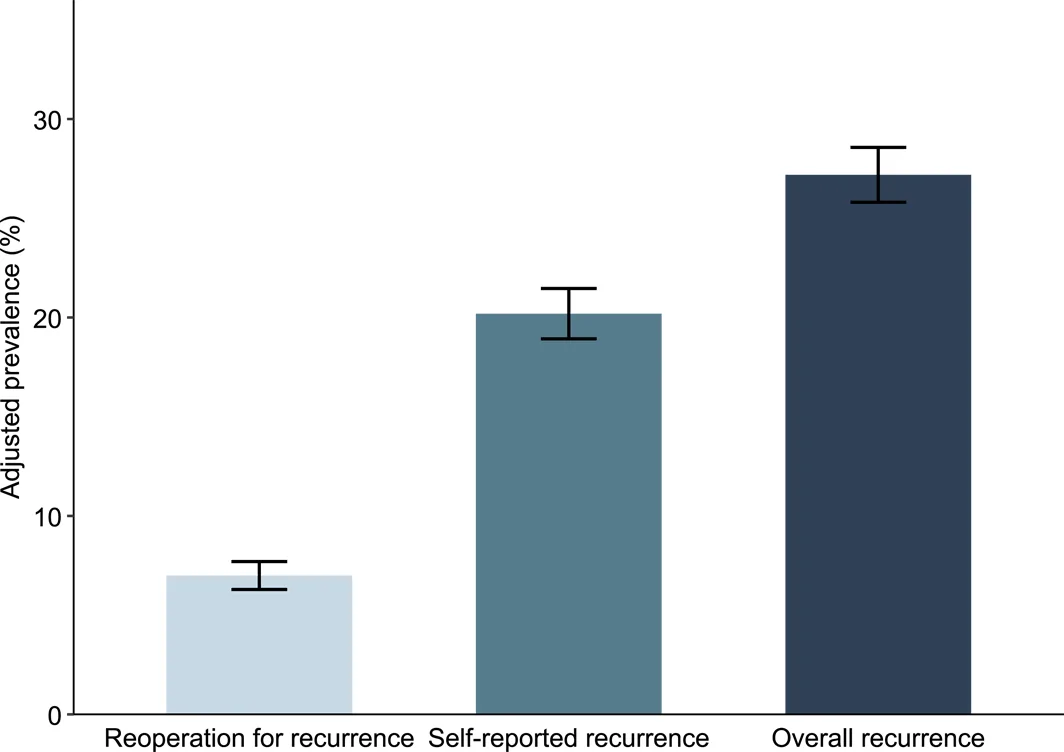
Incisional hernia repair. Adjusted prevalence of reoperation for recurrence, self‐reported recurrence, and overall recurrence (reoperation for recurrence or self‐reported recurrence). The adjusted prevalences were adjusted for sex, age, smoking, body mass index, Charlson Comorbidity Index, use of mesh, hernia defect width, and follow‐up. Error bars indicate 95% confidence intervals.

For incisional hernias, the adjusted prevalence of overall recurrence was 27.2% (95% CI, 25.8–28.6), of which 7.0% (95% CI, 6.3–7.7) had a reoperation and 20.2% (95% CI, 18.9–21.5) had self‐reported recurrence (Figure 3). Thus, approximately one in four patients with incisional hernia recurrence underwent reoperation.

The underlying regression models are presented in Supporting Information S1: Tables 2–5, and all subgroup analyses showed results consistent with the main analysis for each group (Supporting Information S1: Figures 1–2). In the post hoc analysis that included both primary ventral and incisional hernias, the crude reoperation rate for recurrence was 4.3% among responders and 4.0% among non‐responders.

## 4 Discussion

In this nationwide survey‐ and register‐based study, the prevalence of reoperation for recurrence was found to underestimate the overall prevalence of recurrence by approximately fivefold in primary ventral hernia repair and fourfold in incisional hernia repair.

A previous prospective cohort study including 902 patients found that overall recurrence was approximately fourfold higher than reoperation for recurrence in primary ventral hernia repair and fivefold higher in incisional hernia repair [4]. These findings demonstrate the limitations of using reoperation as a proxy for recurrence. In the same study, self‐reported recurrence demonstrated a sensitivity of 0.86 for detecting clinically confirmed recurrence. Additional support for the validity of self‐reported recurrence comes from a prospective study of laparoscopic inguinal hernia repair, which reported high diagnostic accuracy, with a sensitivity of 1.00 and a specificity of 0.86, using a structured telephone interview comprising three questions, combined with a do‐it‐yourself Valsalva maneuver [43]. Together, these findings support the use of self‐reported recurrence as a credible outcome measure. Although sensitivity was not directly assessed in the present study, it is plausible that it falls within the previously reported range. Combining self‐reported recurrence with reoperation for recurrence may therefore provide a more comprehensive estimate of recurrence after ventral hernia repair. In recent years, there has been an increasing emphasis on incorporating PROs into the evaluation of surgical success [12, 44]. This shift underscores the importance of considering not only whether recurrence occurs, but also its impact on the patient. Although recurrence may reflect technical aspects of the surgical procedure, its clinical relevance ultimately depends on its impact on symptoms and quality of life. A recurrence that causes little or no symptoms and does not impair quality of life may therefore not represent a clinically meaningful complication and may not lead to reoperation. Whether a patient undergoes reoperation may also be influenced by factors such as age, comorbidity, symptom severity, and patient preferences. This study was conducted in Denmark, which has a universal, tax‐funded healthcare system with free access to hospital and surgical care independent of personal income or insurance [45]. In this setting, patients who meet clinical indications for reoperation generally undergo further surgery. Consequently, the discrepancy between reoperation for recurrence and overall recurrence may be even greater in healthcare systems without universal coverage or with substantial out‐of‐pocket costs, where financial barriers may limit access to reoperation.

This study has several strengths. It was reported in accordance with RECORD [15] and CROSS [16], ensuring transparency and reproducibility [46]. The nationwide design provides high external validity, supported by a large sample size and an 80% survey response rate. Linkage of multiple nationwide data sources enabled integration of operative details and patient clinical characteristics with a patient‐reported outcome measure. Importantly, the discrepancy between reoperation for recurrence and overall recurrence (reoperation for recurrence or self‐reported recurrence) was observed in a universal, tax‐funded healthcare system, with free access to surgical care [45], minimizing the influence of financial barriers and strengthening the interpretation of the findings. This study also has limitations. First, as this is an observational study, unmeasured confounding cannot be excluded despite adjustment for multiple relevant covariates. Second, self‐reported recurrence was assessed without systematic clinical examination, which may introduce misclassification. However, previous studies suggest that self‐reported recurrence is a reliable outcome measure, with a high sensitivity for detecting clinically confirmed recurrence [4, 43]. In addition, selection bias related to survey participation cannot be excluded, although the crude rate of reoperation for recurrence was similar among responders and non‐responders. Taken together, while self‐reported recurrence cannot replace clinical assessment, it represents a pragmatic and scalable patient‐reported outcome measure in large population‐based studies where systematic clinical evaluation is not feasible.

This nationwide survey‐ and register‐based study demonstrated that reoperation for recurrence substantially underestimated the overall burden of recurrence after ventral hernia repair. These findings are consistent with previous results [4] and extend the evidence using nationwide data spanning more than a decade. The large sample size enabled subgroup analyses stratified by hernia defect widths and among younger patients without comorbidities, all of which yielded similar results. Reliance on reoperation for recurrence as the sole endpoint will therefore systematically underestimate true recurrence rates. Importantly, many patients with recurrence did not undergo further surgery, underscoring that reoperation represents only a subset of recurrences. This raises important questions about how surgical success should be defined. While anatomical recurrence may reflect technical aspects of the procedure, patient‐centered outcomes such as pain, quality of life, functional recovery, and overall satisfaction may be more relevant to patients [12]. Future studies should therefore consider incorporating PROs alongside traditional surgical endpoints to better capture outcomes that matter most to patients after ventral hernia repair.

In conclusion, the prevalence of reoperation for recurrence underestimated the overall prevalence of recurrence by approximately fivefold in primary ventral hernia repair and fourfold in incisional hernia repair. This suggests that register‐based studies investigating reoperation as a proxy for recurrence underestimate the true burden of recurrence.

## Author Contributions

**Evy Á Lakjuni Guttesen:** writing – original draft, conceptualization, methodology, investigation, formal analysis, visualization. **Hugin Reistrup:** writing – review and editing, conceptualization, methodology, investigation, supervision. **Anders Gram‐Hanssen:** writing – review and editing, conceptualization, methodology, investigation. **Jacob Rosenberg:** writing – review and editing, conceptualization, methodology, investigation, supervision. **Jason Joe Baker:** writing – review and editing, conceptualization, methodology, investigation, formal analysis, supervision.

## Funding

The authors have nothing to report.

## Ethics Statement

Informed consent was collected from all patients at the time of survey participation. This study obtained approvals from the Danish Health Quality Institute and the Danish Data Protection Agency (p‐2023‐14805). Ethical approval was not required under Danish legislation for the survey‐ and register‐based components of the study [39, 40]; ethical approval was, however, obtained from the Capital Region of Denmark's Committees on Health Research Ethics (R‐24033183) to access telephone numbers for reminder calls to non‐responders. The data were supplied by the Danish Health Quality Institute and the Danish Health Data Authority (FSEID‐00006834).

## Conflicts of Interest

The authors declare no conflicts of interest.

## Supporting information


Supporting Information S1

## Data Availability

Due to Danish legislation, supporting data are not available.
