# Supplementary material for: Reoperation Versus Self‐Reported Recurrence After Ventral Hernia Repair: A Nationwide Survey‐ and Register‐Based Study
Source: World J Surg. 2026 Jul 28;50(9):2600–8. doi: 10.1002/wjs.70512 (PMC13576578; doi:10.1002/wjs.70512)
Supplement: Supplementary file 1 [file WJS-50-2600-s001.docx]

# SUPPLEMENTAL MATERIAL

**Title:** Reoperation versus self-reported recurrence after ventral hernia repair: a nationwide survey- and register-based study

**Journal:** World Journal of Surgery

**Authors:**

Evy Á Lakjuni Guttesen^1^, MS

Hugin Reistrup^1^, MD, PhD

Anders Gram-Hanssen^1^, MD, PhD

Jacob Rosenberg^1^, MD, DMSc

Jason Joe Baker^1^, MD, PhD

Affiliations:

^1^ Center for Perioperative Optimization, Department of Surgery, Copenhagen University Hospital - Herlev and Gentofte, Borgmester Ib Juuls Vej 1, DK-2730 Herlev, Denmark

**Corresponding author**

Evy Á Lakjuni Guttesen, MS

ORCID ID: (0009-0009-3539-5624)

E-mail: [ealakjuni@gmail.com](mailto:ealakjuni@gmail.com)

**Supplementary Materials**

| **Table S1.** Patient characteristics and operative details for non-responders stratified by hernia type. | *page 3* |
| --- | --- |
| **Table S2.** Multivariable logistic regression analyses with adjusted odds ratios for covariates associated with overall recurrence (reoperation for recurrence or self-reported recurrence) vs no recurrence for primary ventral hernia (umbilical and epigastric). | *page 4* |
| **Table S3.** Multinomial logistic regression analyses with adjusted odds ratios for covariates associated with self-reported recurrence vs no recurrence and reoperation for recurrence vs no recurrence for primary ventral hernia (umbilical and epigastric). | *page 5* |
| **Table S4**. Multivariable logistic regression analyses with adjusted odds ratios for covariates associated with overall recurrence (reoperation for recurrence or self-reported recurrence) vs no recurrence for incisional hernia. | *page 6* |
| **Table S5.** Multinomial logistic regression analyses with adjusted odds ratios for covariates associated with self-reported recurrence vs no recurrence and reoperation for recurrence vs no recurrence for incisional hernia. | *page 7* |
| **Figure S1.** Primary ventral hernia repair. Subgroup analyses of adjusted prevalence for reoperation for recurrence, self-reported recurrence, and overall recurrence (reoperation for recurrence or self-reported recurrence). | *page 8* |
| **Figure S2**. Incisional hernia repair. Subgroup analyses of adjusted prevalence for reoperation for recurrence, self-reported recurrence, and overall recurrence (reoperation for recurrence or self-reported recurrence). | *page 9* |

Table S1. Patient characteristics and operative details for non-responders stratified by hernia type.

|  | | **Primary ventral hernia**  (n = 4244) | **Incisional hernia**  (n = 943) |
| --- | --- | --- | --- |
| Male sex | | 2753 (65) | 405 (43) |
| Age in years, median [IQR] | | 45 [35 – 55] | 53 [43 – 65] |
| Charlson Comorbidity Index | | |  |
|  | 0 | 3346 (79) | 521 (55) |
|  | 1 | 508 (12) | 163 (17) |
|  | 2 | 228 (5) | 151 (16) |
|  | 3 | 92 (2) | 55 (6) |
|  | ≥4 | 70 (2) | 53 (6) |
| Hernia defect width in cm | | |  |
|  | ≤1 | 2384 (56) | 215 (23) |
|  | >1 – 4 | 1750 (41) | 497 (53) |
|  | >4 – 10 | 110 (3) | 231 (24) |
| Suture or mesh placement | | |  |
|  | Suture | 1590 (38) | 127 (14) |
|  | Onlay | 1336 (32) | 238 (25) |
|  | Retromuscular | 144 (3) | 129 (14) |
|  | Preperitoneal | 580 (14) | 106 (11) |
|  | IPOM | 230 (5) | 133 (14) |
|  | IPOM plus | 345 (8) | 199 (21) |
|  | Other^a^ | 19 (<1) | 11 (1) |
| Follow-up in years, median [IQR] | | 2 [1 – 3] | 1 [1 – 3] |
| Reoperation for recurrence | | 141 (3) | 67 (7) |

Data are presented as n (%) unless otherwise indicated. IPOM, intraperitoneal onlay mesh; IPOM plus, intraperitoneal onlay mesh with defect closure; IQR, interquartile range.

^a^ Other includes plug, inlay, and other mesh placements.

Table S2. Multivariable logistic regression analyses with adjusted odds ratios for covariates associated with overall recurrence (reoperation for recurrence or self-reported recurrence) vs no recurrence for primary ventral hernia (umbilical and epigastric).

|  | | **Overall recurrence vs no recurrence** | |
| --- | --- | --- | --- |
|  | | OR (95% CI) | p value |
| Age in years | | 0.98 (0.97 – 0.98) | <0.001 |
| Sex, female | | 1.77 (1.61 – 1.95) | <0.001 |
| BMI in kg/m2 | |  |  |
|  | <18.5 | 0.86 (0.52 – 1.38) | 0.547 |
|  | 18.5–24.9 | Reference | - |
|  | 25 – 29.9 | 1.10 (0.98 – 1.23) | 0.098 |
|  | 30 – 34.9 | 1.43 (1.26 – 1.64) | <0.001 |
|  | 35 – 39.9 | 1.64 (1.37 – 1.97) | <0.001 |
|  | ≥40 | 1.49 (1.16 – 1.90) | 0.002 |
| Smoking | |  |  |
|  | No | Reference | - |
|  | Yes | 1.31 (1.16 – 1.48) | <0.001 |
|  | Previous | 1.11 (1.00 – 1.23) | 0.048 |
| Charlson Comorbidity Index | | |  |
|  | 0 | Reference |  |
|  | 1 | 1.26 (1.11 – 1.44) | <0.001 |
|  | 2 | 1.12 (0.93 – 1.34) | 0.234 |
|  | 3 | 1.37 (0.99 – 1.86) | 0.050 |
|  | ≥4 | 1.65 (1.18 – 2.27) | 0.003 |
| Defect width in cm | | |  |
|  | ≤1 | 0.96 (0.86 – 1.06) | 0.413 |
|  | >1 – 4 | Reference | - |
|  | >4 – 10 | 0.91 (0.68 – 1.20) | 0.526 |
| Non-Mesh | | 1.94 (1.75 – 2.15) | <0.001 |

Models were adjusted for age, sex, BMI, smoking status, Charlson Comorbidity Index, defect size, use of mesh, and follow-up time. Follow-up time was modeled using natural cubic splines and is therefore not presented as odds ratios. BMI, body mass index; OR, Odds ratio; CI, confidence interval.

Table S3. Multinomial logistic regression analyses with adjusted odds ratios for covariates associated with self-reported recurrence vs no recurrence and reoperation for recurrence vs no recurrence for primary ventral hernia (umbilical and epigastric).

|  | | **Self-reported recurrence vs no recurrence** | |  | **Reoperation for recurrence vs no recurrence** | |
| --- | --- | --- | --- | --- | --- | --- |
|  | | OR (95% CI) | p value |  | OR (95% CI) | p value |
| Age in years | | 0.98 (0.97 – 0.98) | <0.001 |  | 0.97 (0.97 – 0.98) | <0.001 |
| Sex, female | | 1.82 (1.64 – 2.02) | <0.001 |  | 1.53 (1.24 – 1.89) | <0.001 |
| BMI in kg/m2 | |  |  |  |  |  |
|  | <18.5 | 0.96 (0.58 – 1.60) | 0.873 |  | 0.51 (0.14 – 1.82) | 0.303 |
|  | 18.5 – 24.9 | Reference | - |  | Reference | - |
|  | 25 – 29.9 | 1.06 (0.93 – 1.20) | 0.374 |  | 1.32 (1.04 – 1.70) | 0.026 |
|  | 30 – 34.9 | 1.37 (1.19 – 1.58) | <0.001 |  | 1.75 (1.31 – 2.35) | <0.001 |
|  | 35 – 39.9 | 1.54 (1.27 – 1.88) | <0.001 |  | 2.23 (1.49 – 3.34) | <0.001 |
|  | ≥40 | 1.37 (1.05 – 1.79) | 0.022 |  | 2.15 (1.24 – 3.72) | 0.006 |
| Smoking | |  |  |  |  |  |
|  | No | Reference | - |  | Reference | - |
|  | Yes | 1.34 (1.18 – 1.53) | <0.001 |  | 1.12 (0.85 – 1.47) | 0.419 |
|  | Previous | 1.09 (0.97 – 1.22) | 0.140 |  | 1.18 (0.94 – 1.47) | 0.159 |
| Charlson Comorbidity Index | | |  |  |  |  |
|  | 0 | Reference | - |  | Reference | - |
|  | 1 | 1.19 (1.04 – 1.37) | 0.015 |  | 1.63 (1.24 – 2.14) | <0.001 |
|  | 2 | 1.09 (0.89 – 1.34) | 0.384 |  | 1.21 (0.81 – 1.81) | 0.341 |
|  | 3 | 1.39 (0.99 – 1.96) | 0.056 |  | 1.28 (0.62 – 2.63) | 0.502 |
|  | ≥4 | 1.66 (1.16 – 2.36) | 0.005 |  | 1.74 (0.82 – 3.70) | 0.152 |
| Defect width in cm | | |  |  |  |  |
|  | ≤1 | 0.97 (0.86 – 1.08) | 0.546 |  | 0.94 (0.75 – 1.18) | 0.612 |
|  | >1 – 4 | Reference | - |  | Reference | - |
|  | >4 – 10 | 1.00 (0.74 – 1.35) | 0.978 |  | 0.57 (0.29 – 1.14) | 0.111 |
| Non-Mesh | | 1.59 (1.42 – 1.78) | <0.001 |  | 4.40 (3.53 – 5.48) | <0.001 |

Models were adjusted for age, sex, BMI, smoking status, Charlson Comorbidity Index, defect size, use of mesh, and follow-up time. Follow-up time was modeled using natural cubic splines and is therefore not presented as odds ratios. BMI, body mass index; OR, Odds ratio; CI, confidence interval.

Table S4. Multivariable logistic regression analyses with adjusted odds ratios for covariates associated with overall recurrence (reoperation for recurrence or self-reported recurrence) vs no recurrence for incisional hernia.

|  | | **Overall recurrence vs no recurrence** | |
| --- | --- | --- | --- |
|  | | OR (95% CI) | p value |
| Age in years | | 0.98 (0.98 – 0.99) | <0.001 |
| Sex, female | | 1.27 (1.09 – 1.48) | 0.003 |
| BMI in kg/m2 | |  |  |
|  | <18.5 | 1.12 (0.44 – 2.63) | 0.807 |
|  | 18.5 – 24.9 | Reference | - |
|  | 25 – 29.9 | 1.03 (0.84 – 1.25) | 0.789 |
|  | 30 – 34.9 | 1.14 (0.92 – 1.42) | 0.228 |
|  | 35 – 39.9 | 1.48 (1.10 – 1.99) | 0.010 |
|  | ≥40 | 1.50 (1.02 – 2.19) | 0.037 |
| Smoking | |  |  |
|  | No | Reference | - |
|  | Yes | 1.28 (1.04 – 1.57) | 0.020 |
|  | Previous | 1.23 (1.04 – 1.46) | 0.017 |
| Charlson Comorbidity Index | | |  |
|  | 0 | Reference | - |
|  | 1 | 1.30 (1.04 – 1.63) | 0.020 |
|  | 2 | 1.24 (1.01 – 1.52) | 0.041 |
|  | 3 | 1.54 (1.13 – 2.08) | 0.006 |
|  | ≥4 | 1.29 (0.95 – 1.74) | 0.103 |
| Defect width in cm | | |  |
|  | ≤1 | 1.03 (0.83 – 1.28) | 0.775 |
|  | >1 – 4 | Reference | - |
|  | >4 – 10 | 1.28 (1.08 – 1.52) | 0.005 |
| Non-Mesh | | 1.60 (1.23 – 2.07) | <0.001 |

Models were adjusted for age, sex, BMI, smoking status, Charlson Comorbidity Index, defect size, use of mesh, and follow-up time. Follow-up time was modeled using natural cubic splines and is therefore not presented as odds ratios. BMI, body mass index; OR, Odds ratio; CI, confidence interval.

Table S5. Multinomial logistic regression analyses with adjusted odds ratios for covariates associated with self-reported recurrence vs no recurrence and reoperation for recurrence vs no recurrence for incisional hernia.

|  | | **Self-reported recurrence vs**  **no recurrence** | |  | **Reoperation for recurrence vs**  **no recurrence** | |
| --- | --- | --- | --- | --- | --- | --- |
|  | | OR (95% CI) | p value |  | OR (95% CI) | p value |
| Age in years | | 0.99 (0.98 – 0.99) | <0.001 |  | 0.97 (0.96 – 0.99) | <0.001 |
| Sex, female | | 1.32 (1.11 – 1.56) | 0.002 |  | 1.08 (0.80 – 1.46) | 0.621 |
| BMI in kg/m2 | |  |  |  |  |  |
|  | <18.5 | 0.61 (0.17 – 2.13) | 0.438 |  | 3.04 (0.83 – 11.11) | 0.093 |
|  | 18.5 – 24.9 | Reference | - |  | Reference | - |
|  | 25 – 29.9 | 0.94 (0.76 – 1.17) | 0.603 |  | 1.46 (0.98 – 2.18) | 0.062 |
|  | 30 – 34.9 | 1.11 (0.88 – 1.41) | 0.386 |  | 1.31 (0.83 – 2.04) | 0.234 |
|  | 35 – 39.9 | 1.51 (1.10 – 2.07) | 0.010 |  | 1.32 (0.68 – 2.58) | 0.409 |
|  | ≥40 | 1.44 (0.96 – 2.17) | 0.080 |  | 1.86 (0.85 – 4.09) | 0.112 |
| Smoking | |  |  |  |  |  |
|  | No | Reference | - |  | Reference | - |
|  | Yes | 1.32 (1.05 – 1.65) | 0.015 |  | 1.03 (0.66 – 1.60) | 0.905 |
|  | Previous | 1.15 (0.95 – 1.39) | 0.146 |  | 1.57 (1.13 – 2.18) | 0.008 |
| Charlson Comorbidity Index | | |  |  |  |  |
|  | 0 | Reference | - |  | Reference | - |
|  | 1 | 1.27 (1.00 – 1.62) | 0.053 |  | 1.32 (0.84 – 2.07) | 0.221 |
|  | 2 | 1.17 (0.93 – 1.47) | 0.183 |  | 1.50 (1.01 – 2.24) | 0.046 |
|  | 3 | 1.60 (1.15 – 2.22) | 0.005 |  | 1.28 (0.67 – 2.44) | 0.459 |
|  | ≥4 | 1.18 (0.84 – 1.66) | 0.345 |  | 1.66 (0.95 – 2.90) | 0.075 |
| Defect width in cm | | |  |  |  |  |
|  | ≤1 | 1.14 (0.90 – 1.44) | 0.289 |  | 0.76 (0.48 – 1.18) | 0.220 |
|  | >1 – 4 | Reference | - |  | Reference | - |
|  | >4 – 10 | 1.19 (0.98 – 1.43) | 0.079 |  | 1.78 (1.27 – 2.51) | <0.001 |
| Non-Mesh | | 1.17 (0.87 – 1.58) | 0.297 |  | 3.86 (2.45 – 6.08) | <0.001 |

Models were adjusted for age, sex, BMI, smoking status, Charlson Comorbidity Index, defect size, use of mesh, and follow-up time. Follow-up time was modeled using natural cubic splines and is therefore not presented as odds ratios. BMI, body mass index; OR, Odds ratio; CI, confidence interval.

Figure S1. Primary ventral hernia repair. Subgroup analyses of adjusted prevalence for reoperation for recurrence, self-reported recurrence, and overall recurrence (reoperation for recurrence or self-reported recurrence).

Analyses were stratified by hernia defect width (≤1 cm, >1–4 cm, and >4–10 cm) and low risk patients (defined as patients aged ≤65 years with a Charlson Comorbidity Index of 0). Error bars indicate 95% confidence intervals. n, number of patients.

Figure S2. Incisional hernia repair. Subgroup analyses of adjusted prevalence for reoperation for recurrence, self-reported recurrence, and overall recurrence (reoperation for recurrence or self-reported recurrence).

Analyses were stratified by hernia defect width (≤1 cm, >1–4 cm, and >4–10 cm) and low risk patients (defined as patients aged ≤65 years with a Charlson Comorbidity Index of 0). Error bars indicate 95% confidence intervals. n, number of patients.
